# How does a local instrumental variable method perform across settings with instruments of differing strengths? A simulation study and an evaluation of emergency surgery

**DOI:** 10.1002/hec.4719

**Published:** 2023-06-11

**Authors:** Silvia Moler‐Zapata, Richard Grieve, Anirban Basu, Stephen O’Neill

**Affiliations:** ^1^ Department of Health Services Research and Policy London School of Hygiene and Tropical Medicine London UK; ^2^ Department of Pharmacy, and Departments of Health Services and Economics The Comparative Health Outcomes, Policy, and Economics (CHOICE) Institute University of Washington Seattle Washington USA; ^3^ National Bureau of Economic Research Cambridge Massachusetts USA

**Keywords:** emergency surgery, instrument strength, instrumental variables, tendency to operate

## Abstract

Local instrumental variable (LIV) approaches use continuous/multi‐valued instrumental variables (IV) to generate consistent estimates of average treatment effects (ATEs) and Conditional Average Treatment Effects (CATEs). There is little evidence on how LIV approaches perform according to the strength of the IV or with different sample sizes. Our simulation study examined the performance of an LIV method, and a two‐stage least squares (2SLS) approach across different sample sizes and IV strengths. We considered four ‘heterogeneity’ scenarios: homogeneity, overt heterogeneity (over measured covariates), essential heterogeneity (unmeasured), and overt and essential heterogeneity combined. In all scenarios, LIV reported estimates with low bias even with the smallest sample size, provided that the instrument was strong. Compared to 2SLS, LIV provided estimates for ATE and CATE with lower levels of bias and Root Mean Squared Error. With smaller sample sizes, both approaches required stronger IVs to ensure low bias. We considered both methods in evaluating emergency surgery (ES) for three acute gastrointestinal conditions. Whereas 2SLS found no differences in the effectiveness of ES according to subgroup, LIV reported that frailer patients had worse outcomes following ES. In settings with continuous IVs of moderate strength, LIV approaches are better suited than 2SLS to estimate policy‐relevant treatment effect parameters.

## 1 INTRODUCTION

The personalization of treatment choice can be informed by comparative effectiveness research that exploits the widespread availability of electronic health records (EHRs), but requires methods that address confounding and heterogeneity. For conventional linear Instrumental Variable (IV) methods, such as two‐stage least squares (2SLS) to identify policy‐relevant estimands such as the Average Treatment Effect (ATE) or Conditional Average Treatment Effects (CATEs), it is required that there is no essential heterogeneity (Heckman et al., 2006). Essential heterogeneity arises when treatment effects differ over levels of unmeasured confounders, in which case 2SLS no longer identifies the ATE, even if the instrument is strong and valid (Heckman et al., 2006). Essential heterogeneity is a major concern in health care, as it is commonly the case that there are biological correlations between risk factors, some of which remain unobserved to the analyst.

In the presence of essential heterogeneity, Local Instrumental Variable (LIV) approaches can provide consistent estimates of the ATE and CATEs (Heckman & Vytlacil, 2005). LIV methods draw on theory about individual's choices to identify ‘marginal treatment effects’ (MTEs) for individuals at the ‘margin of treatment choice’ (Bjorklund & Moffitt, 1983; Heckman & Vytlacil, 1999). These MTEs are identified for individuals for whom the level of the IV is such that observed characteristics encouraging treatment (including the IV) and unobserved characteristics discouraging treatment are balanced, so there is equipoise about the treatment decision. Here, a small change (or nudge) in the level of a valid, continuous IV ‘tips the balance’ for the treatment decision for these marginal patients, without changing the distribution of the underlying risk factors. Therefore, comparing mean outcomes between two groups of patients only separated by a small change in the IV, identifies MTEs for individuals who *comply* with the change in treatment, due to that small change in the IV. A continuous instrument with sufficient support allows all individuals to be defined as ‘compliers’ at some level of the IV (Heckman & Vytlacil, 1999). Hence, given observed covariates, MTEs can be estimated along the continuum of the IV, and aggregated to provide CATEs and ATEs (Heckman & Vytlacil, 1999, 2001, 2005).

The theoretical properties of these LIV methods in settings with essential heterogeneity have been discussed by Heckman et al. (2006), Basu et al. (2007) and Angrist & Fernández‐Val, 2011 inter alia. However, most simulation studies of IV methods only consider treatment effects that are homogeneous, or heterogenous according to measured factors (overt heterogeneity) (Martínez‐Camblor et al., 2019; Terza et al., 2008a, 2008b). Studies that have considered essential heterogeneity, have found that 2SLS provides inconsistent estimates of the ATE (Basu, Coe, & Chapman, 2018; Brooks et al., 2018; Chapman & Brooks, 2016), whereas Basu (2014) reports that a LIV method could provide consistent estimates of the ATE and CATE in finite samples for different types of outcomes. LIV methods have now been applied across a multitude of settings including cardiovascular and bariatric surgery, universal child care programs and transfers to intensive care units (Basu, Jones, & Rosa Dias, 2018; Cornelissen et al., 2018; Grieve et al., 2019; Reynolds et al., 2021).

A major barrier to wider use of potentially valid IVs in general is that if the IV is only weakly associated with treatment assignment, then IV estimators can provide very biased and imprecise estimates (Bound et al., 1995; Nelson & Startz, 1990; Stock & Yogo, 2005). Weak IVs can also amplify the bias arising due to violations of the other assumptions (Bound et al., 1995; Small & Rosenbaum, 2008). While current practice tends to rely on the first‐stage F‐statistic exceeding the value of 10 (Staiger & Stock, 1997), recent developments in the weak identification literature for IV models have revealed the shortcomings of an unequivocal decision rule for assessing weak identification (Andrews et al., 2019; Keane & Neal, 2023; Lee et al., 2021; Moffitt & Zahn, 2022). For LIV to provide consistent, precise estimates of ATE or CATEs, requires a strong continuous/multi‐valued IV with sufficient support to ensure that there is a level of the IV at which each unit ‘complies’ (i.e., is selected into treatment according to the level of the IV). However, no study has assessed the levels of IV strength that are required for an LIV estimator to perform well, nor how performance may differ according to the sample size available, in settings with essential heterogeneity.

This paper addresses this gap in the literature by contrasting LIV with the commonly used 2SLS estimator in Monte Carlo simulations, motivated by a case study which highlights typical issues pertaining to heterogeneity, sample size and IV strength. We simulate four scenarios: two of them under restrictive assumptions about heterogeneity (A: homogeneity; B: overt heterogeneity), one where treatment effects are allowed to be heterogenous according to an unmeasured confounder (C: essential heterogeneity), and one where both forms of heterogeneity are present (D: overt and essential heterogeneity). Across all scenarios, ATE and CATE are the parameters of interest.

This paper is structured as follows. In Section 2, we outline the motivating example. In Section 3, we define the estimands and identification assumptions for 2SLS and LIV and present the methods for the simulation study. In Section 4, we present the results of the simulation study and the case study. In Section 5, we discuss how this study adds to the literature and the implications for further research.

## 2 MOTIVATING EXAMPLE: THE ESORT STUDY

The ESORT (Emergency Surgery OR noT) study evaluated the effectiveness of ES for acute gastrointestinal conditions. The primary outcome of the study was the number of ‘days alive and out of hospital’ (DAOH) at 90‐days (see (Hutchings et al., 2022) for details), which encompasses mortality and total length of hospital stay (LOS). The study exemplifies the key issues that arise when applying IV methods to EHR data to provide policy‐relevant estimates of comparative effectiveness (ESORT Study Group, 2020; Hutchings et al., 2021, 2022). Patients presented as emergency admissions and were selected for either ES, or alternative non‐emergency surgery (NES) interventions such as medical management or delayed surgery, according to unmeasured characteristics such as the severity of the disease, and hence unmeasured confounding and essential heterogeneity were major concerns.

The ESORT study followed Keele et al. (2018) and developed a continuous preference‐based IV for ES receipt to evaluate the effectiveness of ES for three acute gastrointestinal conditions: acute appendicitis, gallstone disease and abdominal wall hernia, using routine hospitalization data from the hospital episode statistics (HES) inpatient database in England. The IV was the hospital's tendency to operate (TTO), a proxy measure of the hospital's latent preference for ES, defined as the proportion of eligible emergency admissions in each of 174 hospitals who had ES in the year preceding each admission. Given a relevant IV, two main assumptions need to hold: (i) conditional on the variables included in the models, the hospital's TTO was not correlated with the patient's outcome except through treatment assignment, (ii) it does not increase the probability of treatment for an individual at some value of the IV, but decrease it for higher values. The study design had some important features to support this assumption. First, in this emergency setting, patients were unlikely to select the hospital according to quality of care. Second, the study only included direct admissions to hospital, so there was no scope to transfer the patient according to physician or patient choice. Third, information was collated on a rich set of proxies for the hospital's quality of acute care, including rates of mortality and emergency admissions in previous years, which were included in the models as fixed effects. Fourth, observed covariates, were balanced across all levels of the TTO, which helped support the requisite assumption that the IV also balanced unmeasured confounders (Hutchings et al., 2022; Moler‐Zapata et al., 2022). The requisite assumption that the IV has a monotonic effect on treatment receipt could not be formally tested on the data. However, it was deemed plausible in this setting, as it seems unlikely that there are patients who would receive ES when admitted to hospitals with low TTO but receive NES when admitted into a hospital with high TTO.

The ESORT study highlighted several outstanding concerns pertaining to IV methods in general, and LIV approach in particular. While the study reported estimates of the ATE, from the outset, there was policy interest in estimating the CATEs, according to baseline covariates including age, number of comorbidities, and levels of frailty. While the sample sizes for each condition, were relatively large, they also differed across conditions, from 268,144 (appendicitis) and 240,977 (gallstone disease), to 106,432 (hernia) patients. There were also differences in the strength of the IV with F‐statistics ranging from 141 (acute appendicitis), 739 (hernia) to 9053 (gallstone disease). Hence, the ESORT study further motivated the interest in what strength of continuous IV was required to provide unbiased, efficient estimates of policy relevant estimands such as CATEs in settings with essential heterogeneity, and according to different sample sizes.

## 3 METHODS

### 3.1 Instrumental variables methods

Throughout we use the Neyman‐Rubin potential outcomes framework (Neyman, 1990; Rubin, 1974). Let Y denote the outcome, D denote the treatment status, and Z denote the IV. Let $D_{Z}$ denote the potential treatment status that would be observed if Z would be set to Z=z, and $Y_{D}$ denote the potential outcome that would be observed if D would be set to D=d, with d∈{0,1}, such that we observe $\left(Y_{D},D_{Z},Z\right)$ for each individual. For each patient, let $Y_{1}=\mu_{1}\left(X_{O},X_{U},\vartheta \right)$ and $Y_{0}=\mu_{0}\left(X_{O},X_{U},\vartheta \right)$ denote the potential outcomes, where $X_{O}$ is the vector of observed covariates, $X_{U}$ is a vector of unmeasured confounders, and ϑ captures all the remaining unobserved random variables. Throughout, we assume exogeneity of the covariates (A1), so that the treatment assignment is the only source of endogeneity, such that $\left(X_{O},X_{U}\right)⊥\vartheta$ and $X_{O}⊥X_{U}$.

#### 3.1.1 Identification assumptions

Angrist et al. (1993) defined a series of structural assumptions for the identification of the LATE. Here, following Abadie (2003) and Tan (2006) we make the following assumptions which are the conditional version of the assumptions outlined by Angrist et al. (1993):

**Table:** 

(A2)	Unconfoundedness of Z	$\left(Y_{d_{z}},D_{z}\right)⊥Z|X_{O}$
(A3)	Exclusion restriction	$Y_{d_{z}}=Y_{d}$ with probability 1
(A4)	Relevance	0<P(Z=z)<1
(A5)	Monotonicity	If $z^{\prime }>z$ then $D_{z^{\prime }}\geq D_{z}$ with probability 1
(A6)	Stable unit treatment value assumption	${D=D}_{Z}$ and ${Y=Y}_{D}$

Assumption (A2) requires that Z is as good as randomly assigned within levels of $X_{O}$. Assumption (A3) rules out the possibility that Z has a direct effect on the outcome other than through $D_{z}$. Assumptions (A2) and (A3) ensure that the only effect of the Z on the outcome is through $D_{z}$. This is sometimes called the independence assumption. Assumption (A4) ensures that Z and $D_{z}$ are correlated conditional on $X_{O}$. Assumption (A5) requires that an increase in Z always results in a higher or equal level of treatment assignment. Assumption (A6) requires that one individual's potential outcomes ($Y_{D}$) and treatments ($D_{z})$ are not influenced by other individuals' levels of Z (i.e., no interference), nor by how the instrument or treatment is delivered (i.e., no different versions of Z or $D_{z}$).

#### 3.1.2 Estimands

Imbens and Angrist (1994) and Angrist et al. (1993) show that, under the assumptions outlined above, the LATE can be defined as ${∆}^{LATE}\left(x_{o},z,z^{\prime }\right)=E\left[Y_{1}-Y_{0}|X_{O}=x_{o},D_{z}<D_{z^{\prime }}\right]$ and is identified by the IV estimand:

$$\frac{E\left[Y|X_{O}=x_{o},Z=z^{\prime }\right]-E\left[Y|X_{O}=x_{o},Z=z\right]}{E\left[D|X_{O}=x_{o},Z=z^{\prime }\right]-E\left[D|X_{O}=x_{o},Z=z\right]}$$

Vytlacil (2002) and Tan (2006) showed that the independence (A2 and A3) and monotonicity assumptions (A5) of the LATE framework are equivalent to those imposed by a non‐parametric selection model, where treatment assignment depends on whether a latent index $\left(\mu_{D}\left(X_{O},Z\right)\right)$ crosses a particular threshold ($X_{U_{D}}$):

$$D_{z}=1\left\{\mu_{D}\left(X_{O},Z\right)\geq X_{U_{D}}\right\}$$

where $X_{U_{D}}$ is a random variable that captures $X_{U}$ and all other factors influencing treatment assignment but not the outcomes. As in Heckman and Vytlacil (1999, 2001), we can rewrite this equation as $D_{z}=1\left\{P\left(X_{O},Z\right)>V\right\}$, where $V=F_{X_{U_{D}}}\left[X_{U_{D}}|X_{O}=x_{O},Z=z\right]$ with V $⊥\left(Z,X_{O}\right)$ and $P\left(x_{O},z\right)=F_{X_{U_{D}}|x_{O},z}\left[\mu_{D}\left(X_{O},Z\right)\right]$ is the propensity for treatment, and F represents a cumulative distribution function. Therefore, for any arbitrary distribution of $X_{U_{D}}$ conditional on $X_{O}$ and Z, by definition V∼Uniform[0,1] conditional on $X_{O}$ and Z. Then, the MTE can be defined as, ${∆}^{MTE}\left(x_{O},p\right)=E\left(Y_{1}-Y_{0}|X_{O}=x_{O},V=v\right)$ and Heckman and Vytlacil (1999, 2001) showed that, under the standard IV assumptions, it can be identified by:

$$\frac{\partial E_{\vartheta }\left(Y|X_{O}=x_{o},Z=z\right)}{\partial p}=E_{\vartheta }\left[\left(Y_{1}-Y_{0}\right)|X_{O}=x_{o},V=v\right]$$

MTEs can be aggregated directly to obtain estimates of the ATE, as shown in Heckman et al. (2006). Basu (2014) showed that MTEs can be used to derive personalized treatment (PeT) effects for each individual that take into account the plausible range of values that V may take for each patient, in addition to their observed covariates, IV and actual treatment assignment (see Section 3.1.3). The rationale for this approach is that the treatment assignment status provides some information on $X_{U_{D}}$. For patients in the treatment group ($D_{z}=1$), the propensity to choose treatment based on $X_{O}$ and Z must outweigh the propensity to choose the comparator strategy based on $X_{U_{D}}$, that is, $P\left(x_{O},z\right)>v$. For patients in the comparator strategy ($D_{z}=0$), the opposite is true. The PeT effect for an individual is obtained by averaging the MTEs corresponding to that individual's level of $X_{O}$ and Z over those values of unobserved variables that are compatible with that patient's treatment assignment. Hence, ${∆}^{PeT}\left(x_{O},p,D\right)=E\left(Y_{1}-Y_{0}|X_{O}=x_{O},P\left(z,x_{O}\right)>v\right)$ for individuals with $D_{z}=1$ and ${∆}^{PeT}\left(x_{O},p,D\right)=E\left(Y_{1}-Y_{0}|X_{O}=x_{O},P\left(z{,x}_{O}\right)<v\right)$ for individuals with $D_{z}=0.$

All of the treatment effect estimands, including ATE and CATEs, can be derived by appropriately aggregating the PeT effects since these are defined at the individual level (see Section 3.1.3).

#### 3.1.3 Estimation methods

##### Two‐stage least squares estimator

2SLS is a common approach to the implementation of IV methods that consistently estimates the ATE parameter under homogeneity, or the LATE parameter under essential heterogeneity given a binary IV. Under assumptions (A1)‐(A6), the 2SLS (Wald) estimator involves: (i) estimating $E\left[D_{Z}|X_{O},Z\right]$ by regressing $D_{z}$ on $X_{O}$ and Z, and (ii) estimating $E\left[Y_{D}|{D_{z},X}_{O},Z\right]$ by regressing on $X_{O}$ and $\hat{E}\left[D_{Z}|X_{O},Z\right]$. When the instrument is continuous, 2SLS reports a weighted average of LATEs, which requires careful interpretation (Baiocchi et al., 2014).

##### Local Instrumental Variables estimator: Estimating PeT effects

Basu (2014, 2015) describe in detail the series of steps required to estimate PeT effects using the LIV methodology. Briefly, $D_{z}$ is regressed on Z and $X_{O}$, as above, using appropriate methods for binary outcomes and the propensity for treatment $p\left(x_{O},z\right)$ is estimated. Next, Y is regressed on $X_{O}$ and a function of $\hat{p}\left(x_{O},z\right)$ including interactions with $X_{O}$. The approach outlined in Basu (2014) involves differentiating the outcome model g(Y) by $\hat{p}\left(x_{O},z\right)$. Next, PeT effects for each individual can be obtained by performing numerical integration, with MTE $\left(\partial \hat{g}\left(Y\right)/\partial \hat{p}\right)$ evaluated by replacing $\hat{p}$ using 1000 random draws of $u∼unif\left(\min\left(\hat{p}\left(x_{O},z\right)\right),\max\left(\hat{p}\left(x_{O},z\right)\right)\right)$. Then, $D^{*}={﻿\Phi }^{-1}\left\{\hat{p}\left(x_{O},z\right)\right\}+{﻿\Phi }^{-1}\left(1-u\right)$ can be computed. Personalized treatment effects can be computed by averaging $\partial \hat{g}\left(Y\right)/\partial \hat{p}$ over values of u for which $D^{*}>0$ if D=1; or over values of $D^{*}\leq 0$ if D=0. Finally, averaging PeT effects over all of the observations provides an estimate of the ATE for the population, and over strata of $X_{O}$ gives the CATE for the subpopulation of interest. Standard errors can be computed using bootstrap methods (Basu, 2015). We now consider the design of the simulation study to contrast the relative performance of the LIV and 2SLS approaches.

### 3.2 Simulation study

Motivated by the gaps in the extant literature, and the motivating example, this simulation study was designed to consider the relative performance of 2SLS and LIV approaches across settings that differed with respect to the form of heterogeneity, the sample size and the strength of the IV. We report the performance of the methods in a Monte Carlo Simulation study according to their mean bias (%) and Root Mean Squared Error (RMSE) for each estimand (ATE and CATE).

#### 3.2.1 Data generating process

We create 5000 datasets each containing *N* ={5000,10000,50000} units, of which 50% are assigned to the treated group. The data generating process (DGP) includes one observed ($X_{O}$) and one unmeasured ($X_{U}$) covariate. We draw $X_{O}$, $X_{U}$ and the instrument, Z from normal distributions with mean 0, and standard deviation 3. Three subgroups of interest are defined by whether the individuals' values for $X_{O}$ are more than 0.5 standard deviations below or above its mean.

##### Treatment model

The treatment assignment is determined by the latent variable $D^{*}$, defined as:

$$D^{*}=\delta_{D}+3X_{O}-3X_{U}+\delta_{Z}Z+\left(4-\delta_{Z}\right)\epsilon_{D}$$

where $\epsilon_{D}$ has a normal distribution with mean 0 and standard deviation, 1. Treatment is then determined as D=1 if $D^{*}>0$ and D=0 otherwise. The parameters $\delta_{Z}$ and $\delta_{D}$ are chosen to ensure the average F‐statistic, $F_{IV},$ across the datasets equals the desired level $F_{Target}=\left\{10,25,50,100,500,1000\right\}$. $F_{IV}$ is the Cragg and Donald (1993) F‐statistic computed in each dataset as,

$$F_{IV}=\left(N-df_{m}-1\right)*\frac{\sigma_{no\;IV}^{2}-\sigma_{IV}^{2}}{\sigma_{IV}^{2}}$$

where $\sigma_{no\;IV}^{2}$ and $\sigma_{IV}^{2}$ indicate the residual variance from regressing D on $X_{O}$ in a model without interactions with or without including the IV respectively, and $df_{m}$ is the number of parameters in the model excluding the IV (i.e., $df_{m}=2$ here). For a given F‐statistic, a larger sample size implies a lower compliance rate, which in turn will imply a weaker instrument. At low compliance rates, the RMSE of IV estimates can increase substantially (Little et al., 2009). We estimate the compliance rate for each sample size and F‐statistic, by contrasting treatment uptake at the 1^st^ and 99^th^ percentiles of the IV.

##### Outcome model

The outcome models under treatments ($Y_{1}$) and control ($Y_{0})$ can be written as:

$$Y_{0}=\beta_{0}+\beta_{1}X_{O}+\beta_{2}X_{U}+\epsilon_{Y_{0}}$$

$$Y_{1}=\left(\beta_{0}+\tau_{0}\right)+\left(\beta_{1}+\tau_{1}\right)X_{O}+\left(\beta_{2}+\tau_{2}\right)X_{U}+\epsilon_{Y_{1}}$$

Implying the treatment effect is $\tau =E\left(Y_{1}-Y_{0}\right)=\tau_{0}+\tau_{1}X_{O}+\tau_{2}X_{U}$. Specifically we define the outcome under control as follows:

$$Y_{0}=-10-10X_{O}+10X_{U}+N(0,1)$$

We consider 4 scenarios for the outcome under treatment, $Y_{1}$. In Scenario A, effects are homogeneous (τ=50). In Scenario B, effects are heterogeneous but depend only on observed confounders (overt heterogeneity) ($\tau =40+20X_{O}$). In Scenario C, $X_{U}$ influences both the treatment assignment and the gains from treatment ($\tau =40+20X_{U}$). In this Scenario, there is essential heterogeneity but no overt heterogeneity. Finally, in Scenario D there is both overt and essential heterogeneity ($\tau =20+20X_{O}$ $+\;20X_{U}$). Table 1 displays the parameter values for each scenario. The parameter combinations of interest consist of combinations of n={5000,10000,50000} and *F*_*Target*_ ={10,25,50,100,500,1000}.

**TABLE 1:** Definition of the simulation scenarios.

Scenario	Sample size	Target F‐statistic	$\tau_{0}$	$\tau_{1}$	$\tau_{2}$
Scenario A: Homogeneity	All sample sizes $\left(n=\left\{5000,10000,50000\right\}\right)$	All target F‐statistic values $\left(F_{\mathrm{Target}}=\{10,25,50,100,500,1000\}\right)$	50	0	0
Scenario B: Overt heterogeneity			40	20	0
Scenario C: Essential heterogeneity			40	0	20
Scenario D: Overt and essential heterogeneity			20	20	20

*Note:* For each particular scenario, across all sample sizes and target F‐statistic values, the form of treatment effect heterogeneity is defined by the values of $\tau_{0}$, $\tau_{1}$ and $\tau_{2}$.

For each parameter combination for each scenario, we create 5000 datasets using the DGP described above and estimate the treatment effects as described below.

#### 3.2.2 Implementation of methods

For the 2SLS model, we control for $X_{O}$ and instrument D by Z. To capture heterogeneity, we also include an interaction between $X_{O}$ with D, and instrument this with interactions of Z and $X_{O}$. To obtain effect estimates, we use the recycled predictions approach, whereby the two potential outcomes ($Y_{0}\;and\;Y_{1}$) are predicted from the second stage model after setting D=0 or D=1 and the interaction $X_{O}$* $D=0\;or\;X_{O}$ (Basu & Rathouz, 2005). The individual level effect is then estimated as $\hat{\tau }={\hat{Y}}_{1}-{\hat{Y}}_{0}$, allowing us to calculate the ATE, and CATEs for the three subgroups (CATE_1,_ CATE_2,_ and CATE_3_).

For the LIV approach, we first estimate the propensity for treatment conditional on $X_{O}$ and Z, and in the second stage outcome model we include $X_{O}$, D, the estimated propensity score, $\hat{p}$, $\hat{p}$* $X_{O}$ and $\hat{p}$ ^2^. We then estimate PeT effects for each individual as described in Basu (2015) using the petiv command in Stata. The estimated PeT effects are then aggregated to obtain estimates of the ATE, CATE_1,_ CATE_2,_ and CATE_3._ Before applying either method, we remove observations at those levels of the estimated propensity score where there is insufficient overlap (Basu, 2015).

## 4 RESULTS

### 4.1 Simulation study

**FIGURE 1:**
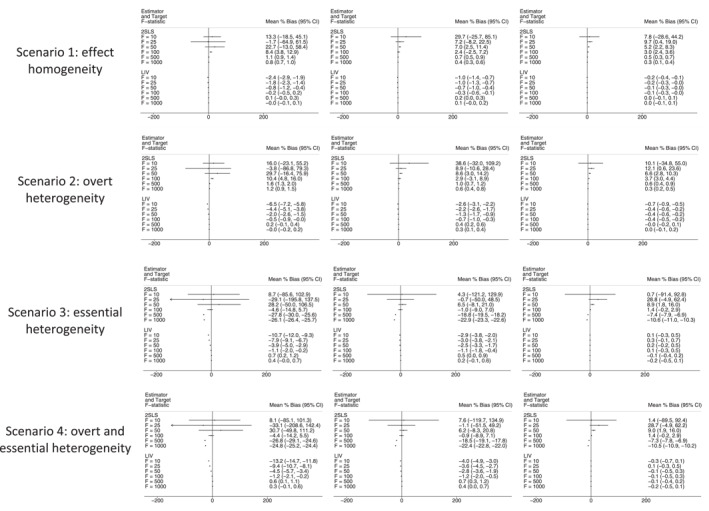
Bias plot for Average Treatment Effect (ATE) estimates across scenarios, with sample sizes of 5000 (left), 10,000 (middle) and 50,000 (right). 2SLS, Two‐Stage Least Squares; CI, Confidence Interval; LIV, Local Instrumental Variables.

**FIGURE 2:**
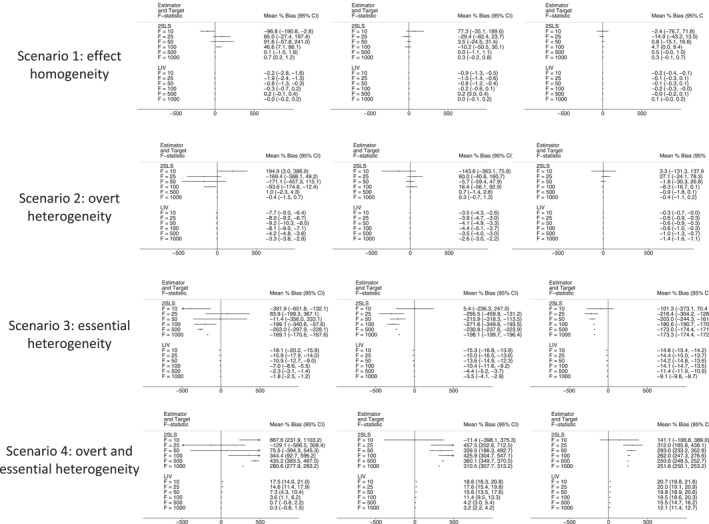
Bias plot for Conditional Average Treatment Effect (CATE) estimates across scenarios, with sample sizes of 5000 (left), 10,000 (middle) and 50,000 (right). 2SLS, Two‐Stage Least Squares; CI, Confidence Interval; LIV, Local Instrumental Variables.

**FIGURE 3:**
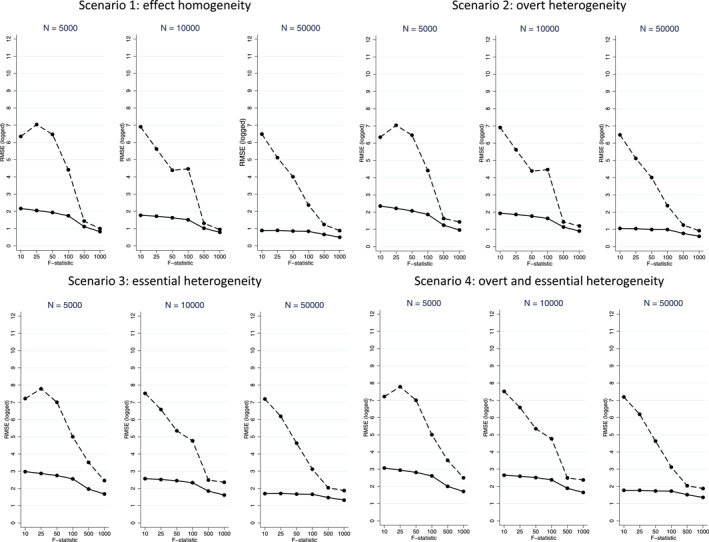
Root Mean Squared Error (RMSE) plots for Average Treatment Effect (ATE) estimates from 2SLS (dashed line) and LIV (solid line) across the scenarios, with sample sizes (*N*) of 5000 (left), 10,000 (middle) and 50,000 (right).

**FIGURE 4:**
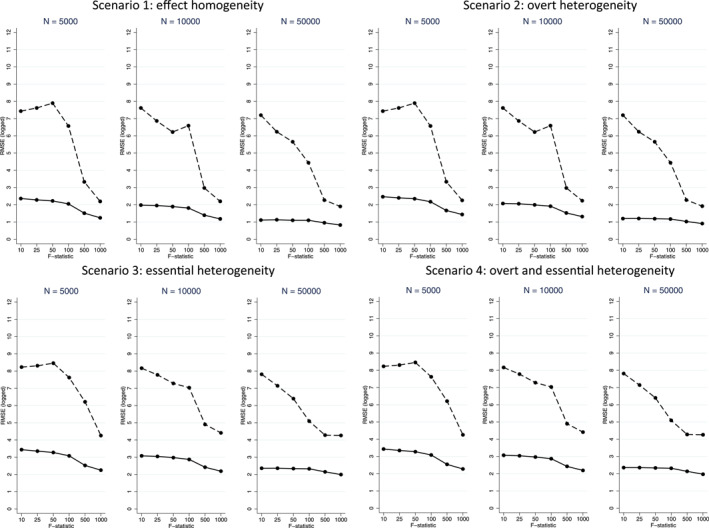
Root Mean Squared Error (RMSE) plots for Conditional Average Treatment Effect (CATE) estimates from 2SLS (dashed line) and LIV (solid line) across the scenarios, with sample sizes (*N*) of 5000 (left), 10,000 (middle) and 50,000 (right).

Figures 1, 2, 3, 4 present mean (%) bias in the ATE and CATE estimates (Figures 1 and 2, respectively) and the corresponding plots for RMSE (Figures 3 and 4, respectively). The results for the three subgroups showed similar patterns, and hence, for brevity, we only report the results for one of them.

In settings with homogenous treatment effects, or with overt heterogeneity, levels of bias in the ATE estimates were generally low (<5%). When the F‐statistic was below 50 or the sample size was smaller (*n* = 5000), the bias for the 2SLS estimates was somewhat higher (5%–10%) (Figure 1). In settings with essential heterogeneity, 2SLS reports relatively high (>10%) levels of mean bias across almost all combinations of IV strength and sample size. The levels of mean bias are only similar between the methods when the target F statistic is high (>100). For 2SLS, the confidence intervals (CI) around the estimates of mean bias are generally wide. For 2SLS to provide estimates with moderate to small levels of bias, with narrow CI around those estimates required an F statistic of at least 100 and a sample size of 50,000. The LIV estimator reports low levels of bias in ATE estimates across all scenarios, aside from those with both a smaller sample size (*n* = 5000) and a F‐statistic of 25 or less (Figure 1).

The bias plots for the CATE estimates have a somewhat similar pattern, although for this estimand the 2SLS estimator reports high levels of mean bias even in settings with overt heterogeneity, unless the sample size is relatively large (*n* = 50,000) and/or the F‐statistic is above 100 (Figure 2). The LIV estimator reports lower levels of bias than 2SLS across the majority of scenarios.

In general, for both methods, across most scenarios, for a given sample size, the levels of mean (%) bias decrease at higher levels of the F‐statistic (Figure 2). The RMSE in the estimates of the ATE are substantially lower for the LIV than the 2SLS estimator, except for those settings with an F‐statistic of 500 or 1000 (Figure 3).^1^ For the CATE, in general, the RMSE estimates mirror the bias results, in that they are substantially lower across all settings for LIV (Figure 4).

Compliance rates for a given F‐statistic were sensitive to the sample size available (see Table 2 below). For a sample size of 5000, increasing the F‐statistic from 10 to 1000 increases the compliance rate from 8% to 73%, while for a sample size of 50,000, the compliance rate only increases from 3% to 29%.

**TABLE 2:** Compliance rate by sample size (*N*) and F‐statistic.

Target F‐statistic	*N* = 5000	*N* = 10,000	*N* = 50,000
10	8%	6%	3%
25	13%	9%	5%
50	18%	13%	6%
100	26%	20%	9%
500	56%	42%	21%
1000	73%	57%	29%

*Note*: Each cell shows the compliance rate for each value of the Target F‐statistic calculated as the difference in treatment probability between highest and lowest quintile of the instrument, Z.

### 4.2 Case study

#### 4.2.1 Case study: Implementation of 2SLS and LIV approaches

LIV estimated PeT effects of ES versus NES on DAOH at 90 days, for each individual allowing for treatment effect heterogeneity and confounding. These PeT effects were aggregated to report the effects of ES overall, and for each pre‐specified subgroup of interest. Since DAOH at 90 days was left skewed due to the maximum being 90 days, we rescaled this to lie between 0 and 1 (90‐DAOH)/90) and effects were then rescaled back to the original scale. Probit regression models were used to estimate the initial propensity score (first stage), while GLMs were applied to the outcome data, with the most appropriate family and link function chosen according to RMSE, with Hosmer‐Lemeshow and Pregibon tests also used to check model fit and appropriateness (Hosmer & Lemeshow, 2000; Pregibon, 1980). The logit link and binomial family were selected for all three conditions. Models at both stages adjusted for baseline measures, time period, and proxies for hospital quality, defined by rates of emergency readmission and mortality in 2009–10 (time constant), and in the year prior to the specific admission concerned (time‐varying).

Estimates of mean differences in DAOH between the comparison groups, overall and for pre‐specified subgroups (CATEs) were reported with standard errors and CI obtained with the non‐parametric bootstrap (300 replications), allowing for the clustering of individuals within hospitals. The 2SLS approach used the same model specification and selection (including covariates used for confounding adjustment) to report estimates overall and for subgroups.

#### 4.2.2 Case study: Results

**FIGURE 5:**
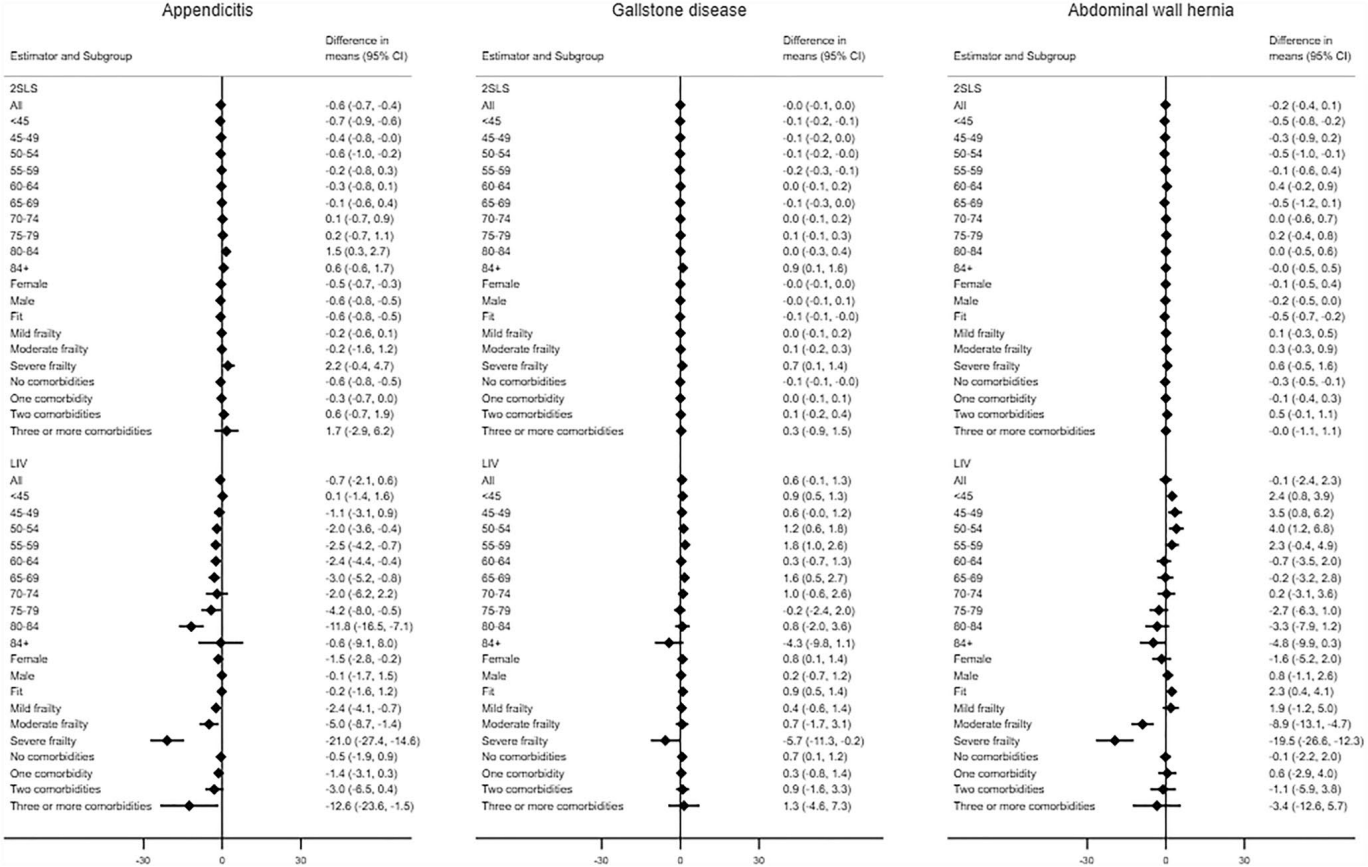
Mean differences in days alive and out of hospital (DAOH) between emergency surgery (ES) and non‐emergency surgery for appendicitis (left), gallstone disease (center) and hernia (right) subgroups. 2SLS, two‐stage least squares; CI, Confidence Interval; LIV, Local Instrumental variables.

The study reported somewhat similar that for both methods the 95% CIs surrounding the mean differences included zero (Figure 5). Beneath this overall result, the LIV approach reported evidence that the effectiveness of ES was heterogeneous according to pre‐specified subgroups. In particular, for all three conditions, ES led to lower DAOH for patients who had severe levels of frailty, and for those with acute appendicitis, ES was less effective for older patients (aged 80–84) or those with three of more comorbidities. By contrast, the 2SLS approach, which failed to account for unobserved heterogeneity (e.g., disease severity), did not report any substantive differences in relative effectiveness according to patient subgroup (Figure 5).

## 5 DISCUSSION

This paper formally assessed the performance of the LIV methodology developed by Heckman and Vytlacil (1999, 2001) and further extended by Basu (2014) to provide policy relevant estimates of ATE and CATE in settings that differed according to the form of heterogeneity, the sample size, and level of IV strength. We contrasted the performance of LIV with that of the widely‐used 2SLS approach. The scenarios considered in the simulation study were directly motivated by gaps in the literature and by a comparative effectiveness study that used LIV in evaluating ES for three acute gastrointestinal conditions for subgroups of prime policy relevance. In the case study, overt and essential heterogeneity were important concerns, amid differing levels of IV strength and sample sizes, and these issues motivated the scenario of prime interest for the simulation study (Scenario D). However, we also considered scenarios, which can, in principle provide accurate estimates of ATE and CATEs with conventional IV methods such as 2SLS (Scenarios A and B). We compared the performance of the two methods, according to bias and statistical efficiency (RMSE).

Four preliminary findings of the simulation study are worth emphasizing. First, our results suggest that while LIV performs better according to increasing levels of IV strength and sample size, this estimator reports relatively low levels of bias in estimates of the ATE and CATEs across all scenarios including those with essential heterogeneity. These findings compliment those of Basu (2014) in evaluating the reliance of the estimator on the relevance condition as well as the consistency of the estimator, but also by considering a wider range of assumptions about heterogeneity.

Second, our results suggest that 2SLS reports biased estimates of the ATE and CATEs in the presence of essential heterogeneity (Scenarios C and D), except in those cases where the instrument is very strong (F‐statistic >500), and sample size is large (*n* = 50,000). These results are consistent with previous findings that 2SLS estimates cannot generally be extrapolated to broader populations beyond the compliers unless restrictive assumptions are made about the heterogeneity of treatment effects (Brooks et al., 2018; Chapman & Brooks, 2016). In scenarios where essential heterogeneity is absent (scenarios A and B), 2SLS reports biases larger than 5% at levels of *F* < 50, unless the sample size is very large (*n* = 50,000). It should be noted that at those values of F the distribution of the mean bias is quite skewed, and this is reflected in the wide CIs around the estimates of mean bias (see Figures 1 and 2). These findings suggest that, in studies without essential heterogeneity, and with large samples and a sufficient strong IV, 2SLS is a simple alternative to LIV.

As the sample size increases the magnitude of the bias is reduced for both methods, but for 2SLS an F statistic of at least 50 (depending on the sample size) is required for estimates of mean bias and the accompanying CI to be <5%. This finding lends support to existing guidance suggesting that the requisite magnitude of the F statistic depends on other factors such as the sample size (Hirano & Porter, 2015; Keane & Neal, 2023). This finding further emphasizes the inadequacy of guidance resting solely on a ‘rule of thumb’ for a single setting, the target F‐statistic, and highlights the importance of these wider considerations of the likely form of heterogeneity, and sample size, as well as the F statistic when interpreting a study's results.

Thirdly, while 2SLS can reliably estimate CATEs in the presence of effect homogeneity or overt heterogeneity given a sufficiently strong IV or large enough sample, in the presence of essential heterogeneity, as theory would suggest, 2SLS can give extremely biased estimates of CATEs, and so in settings where essential heterogeneity is anticipated, 2SLS should not be used to estimate CATEs. In contrast, the LIV method provided estimates with low bias in the presence of overt and/or essential heterogeneity, provided the F‐statistic was greater than 50. Interestingly, for the estimates of the CATEs, we find that as the sample size increases, an increase in the F‐statistic is less beneficial in mitigating bias and reducing RMSE, in line with the observation that a given increase in the F‐statistic has less impact on compliance rates at larger sample sizes.

Finally, LIV generally reported lower levels of RMSE than 2SLS, in particular for estimating the CATEs. However, it is important to note that here the propensity score and outcome models underlying the LIV method are correctly specified, and that performance may deteriorate where this is not the case. Data adaptive approaches could prove useful where model specification is not known.

The findings from the simulation study are informative in interpreting the CATE estimates in the ESORT study. The results offer reassurance that in such settings where essential heterogeneity would appear inevitable, that a LIV approach can provide unbiased estimate of policy‐relevant estimands such as CATE, with sample sizes and F‐statistics smaller than those of the ESORT study. Here, the LIV approach was able to report relative effectiveness according to subgroup, and the finding that for patients with high levels of frailty ES was not cost‐effective (or cost‐effective) is of potential importance. Notwithstanding inevitable concerns about multiple testing, and it should be noted that no formal adjustment for this was made, it is of potential interest for policy, that the finding that the intervention was not cost‐effective for the subgroup with severe frailty was replicated across all five conditions in the original study. Further research to test this hypotheses in different settings is now warranted (Moler‐Zapata et al., 2022).

This study has several strengths. First, it builds on insights and hypotheses raised by a large observational study using EHRs from England. The ESORT study illustrates the main challenges of using LIV methods for comparative effectiveness research and its findings in relation to IV strength, sample size requirements directly informed the scenarios considered in the simulation study. Second, while the uptake of LIV methods has been limited almost entirely to settings with essential heterogeneity, the simulation study considers different forms of heterogeneity of treatment effects as well as the scenario where treatment effects are assumed to be homogeneous in the study population. Future work will expand the simulation study to incorporate other well‐known issues of IVs methods, including the challenges in applying IV estimation methods to non‐linear outcome data (Clarke & Windmeijer, 2010; Vansteelandt et al., 2011). Previous research has shown that the power of 2SLS conveyed by conventional F‐statistic values is low (Keane & Neal, 2023; Y. Lee et al., 2020). In this future work, we will therefore consider the implications of sample size and instrument strength for the power of LIV analyses and confidence interval coverage. Future work will also formally assess whether imbalances in treatment assignment rates are detrimental to consistency and power of LIV inferences. This is an important concern for applied work using EHRs. For instance, the observed difference in the prevalence of ES and NES in ESORT (90/10 in the cohort with appendicitis) could reduce the power of the analysis (Walker et al., 2017). Finally, the current implementation of LIV requires a continuous IV which may not be available in some settings, and remains unknown the extent to which the method may produce biased or inefficient estimates if the underlying assumptions with respect to the treatment assignment or outcome models are violated. Further research can build on the simulation study described to assess these potentially important issues for practice.

## CONFLICT OF INTEREST STATEMENT

The authors declare no conflict of interest.

## ETHICS STATEMENT

The research was approved by the London School of Hygiene and Tropical Medicine ethics committee (Ethics Reference no: 21687).

## Data Availability

The data that support the findings of this study are available from NHS Digital but restrictions apply to the availability of these data, which were used under license for the current study, and so are not publicly available.

## ENDNOTE

^1^
For example, in scenario C, for a level of IV strength when *N* = 5000 and *F* = 10, the logged RMSE for the ATE is 2.98 for LIV and 7.91 for 2SLS. These findings correspond to values of a normalised RMSE, which relates the (unlogged) RMSE to the true ATE, of 0.49 and 67.96 for LIV and 2SLS, respectively.
